# Assessment of the effectiveness and satisfaction of platelet-rich plasma compared with hyaluronic acid in knee osteoarthritis at minimum 7-year follow-up: A *post hoc* analysis of a randomized controlled trial

**DOI:** 10.3389/fbioe.2022.1062371

**Published:** 2022-11-25

**Authors:** Zhengming Wang, Rui Wang, Sicheng Xiang, Yong Gu, Ting Xu, Hengkai Jin, Xinbo Gu, Peijian Tong, Hongsheng Zhan, Shuaijie Lv

**Affiliations:** ^1^ Shi’s Center of Orthopedics and Traumatology, Shuguang Hospital Affiliated to Shanghai University of Traditional Chinese Medicine, Shanghai, China; ^2^ Institute of Traumatology and Orthopedics, Shanghai Academy of Traditional Chinese Medicine, Shanghai, China; ^3^ Zhejiang Chinese Medical University, Hangzhou, Zhejiang, China; ^4^ Zhangjiagang TCM Hospital Affiliated to Nanjing University of Chinese Medicine, Zhangjiagang, Jiangsu, China; ^5^ The First Affiliated Hospital of Zhejiang Chinese Medical University, Zhejiang Provincial Hospital of Chinese Medicine, Hangzhou, Zhejiang, China

**Keywords:** osteoarthritis, platelet-rich plasma, hyaluronic acid, knee, intra-articular injection

## Abstract

**Background:** Knee osteoarthritis (KOA) can be effectively treated conservatively using platelet-rich plasma (PRP) injections into the affected joints. While the short-term therapeutic clinical benefits were well documented, the mid-term results remain undetermined. To clarify its efficacy, the mid-term clinical outcomes of intra-articular injections of either PRP or hyaluronic acid (HA) in KOA were compared.

**Methods:** One hundred patients who complied with the inclusion criteria were randomized to undergo once a week 3 weeks, intra-articular injections of either PRP or HA. Patients were evaluated before the injection, at 3, 6, and a mean of 78.9 months of follow-up. Eighty-five patients reached the final evaluation. Data on survival, re-intervention, pain, function, imaging, and satisfaction were collected and analyzed.

**Results:** With surgery for any reason as the endpoint, the cumulative survival rate of the PRP group was 90%, while that of the HA group was 74%. There was a significant difference between the two groups in the total re-intervention rate (56.7% vs 16.2%, *p* < 0.05). The comparative analyses showed significant intergroup differences in the visual analog scale (VAS) and the Western Ontario and McMaster Universities Arthritis Index (WOMAC) (*p* < 0.01, *p* < 0.05, respectively) at the final follow-up. And base on the regression analyses, the type of treatment, age, and Kellgren-Lawrence (K-L) grade served as statistically an independent determinants of VAS (*p* < 0.001, *p* = 0.034, *p* < 0.001, respectively). Likewise, those variables independently determined WOMAC in our study. However, no difference was observed in the imaging evaluation, containing the K-L grade and Cartilage Lesion Score, between the two groups (*p* > 0.05). Besides, the satisfaction treated by the PRP was 78.6%, with a superiority compared with HA (55.8%, *p <* 0.05), and no complications were noted in the whole treatment process among patients who participated.

**Conclusion:** PRP was more effective than HA in survival and re-intervention rates, VAS, and WOMAC, although there were no significant differences in the imaging evaluation between the two groups. Furthermore, patients treated with PRP were associated with higher satisfaction compared with HA.

## Introduction

On account of the limited regenerative capacity of the articular cartilage (Li et al., 2021), osteoarthritis (OA) is an incurable and crucial disease in the field of orthopedics, of which prevalence is increasing year by year with the effects of aging in the global population (Zhang et al., 2021). It was suggested that 250 million people are currently affected all over the world (Hunter and Bierma-Zeinstra, 2019). Clinically, knee osteoarthritis (KOA), the most common type of osteoarthritis (Prieto-Alhambra et al., 2014), can be successfully managed with conservative interventions in the early stage (Bannuru et al., 2019; Kolasinski et al., 2020; Sharma, 2021), including lifestyle changes, physical therapies, oral drugs, and intra-articular therapies (Deyle et al., 2020; Belk et al., 2021; Gazendam et al., 2021). It is generally acknowledged that the most favorable treatment option for end-stage KOA is arthroplasty which should not be performed too early. For this reason, the main objective of treatment for mild and moderate KOA is to alleviate symptoms and postpone or even stop functional deterioration as long as feasible (Chen et al., 2021). Among several conservative treatment measures, it is noteworthy that intra-articular injection therapies, which involve the injections of various drugs directly into the joints, seem to offer a promising approach to the management of patients with early-stage KOA (Bauer et al., 2022). Glucocorticoids, hyaluronic acid (HA), and mesenchymal stem cells (MSCs) were the widespread medications employed intra-articular injections, while the practice remains controversial. It was reported that there were some adverse joint findings have been structurally observed in patients after glucocorticoid injections (Kompel et al., 2019). The HA plays a role in increasing viscoelastic properties of the synovial fluid and overall joint lubrication in the injured region through its unique physicochemical properties and molecular structure, and its inferiority is lower effective in pain relief and functional improvement (Dai et al., 2017; Bowman et al., 2018). The MSCs secrete various cytokines that modulate an anti-inflammatory milieu in the OA joint and may also have a unique ability to induce the growth of new cartilage-like cells, which gives them immunomodulatory characteristics and makes them a suitable candidate for use in knee cartilage repair (Al-Najar et al., 2017; Lu et al., 2020; Dulic et al., 2021). Whereas the selection of the appropriate donor source and the optimal dose has become an essential issue (Shoukrie et al., 2022). Consequently, a new therapeutic option needs to focus on balancing the risks and benefits of KOA.

Autologous platelet-rich plasma (PRP), the processed liquid fraction of autologous peripheral blood, has been used in various medical fields for more than 30 years, which is characterized by a platelet concentration above the baseline (Marx, 2001; Everts et al., 2020), the release of growth factors (GFs), and promoting concentrated anti-inflammatory signals (Sampson et al., 2010). It has the advantages of low-cost, convenient preparation, and abundant raw materials (Filardo et al., 2011; Hong et al., 2021), moreover of which the short-term clinical benefits have been confirmed by many studies (Sánchez et al., 2022; Wang et al., 2022; Wu et al., 2022) in the early and middle-stage treatment of KOA. However, the medium-to-long term outcomes of intra-articular PRP injections are undetermined. Therefore, we conducted this study to elucidate the medium-term effects of intra-articular PRP injections on clinical symptoms and radiology in patients with KOA.

## Patients and methods

### Patient selection and treatment

This study was a medium-term follow-up of a previous randomized controlled trial (Lyu et al., 2016) which was registered and approved by the Ethics Committee. The informed consent form was obtained from each patient who agreed to complete the follow-up survey. Patient selection, randomization method, and treatment approaches were outlined in a previous publication at length. Briefly, the inclusion criteria were as follows: (1) age between 35 and 85 years; (2) diagnosis consistent with the standard of KOA (Hochberg et al., 1995); (3) Kellgren-Lawrence (K-L) grade I-III(Kellgren and Lawrence, 1952); (4) the normal hematological examination results. Exclusion criteria were adopted: (1) the presence of diabetes, hematological or cardiovascular disease and other systemic diseases, and infections; (2) hemoglobin level lower than 11 g/dl and platelet count less than 150,000/mm^3^; (3) the use of Non-steroidal anti-inflammatory drugs (NSAIDs) within 2 weeks before treatment; (4) Taking anticoagulant drugs and immunosuppressants within 3 months.

**FIGURE 1:**
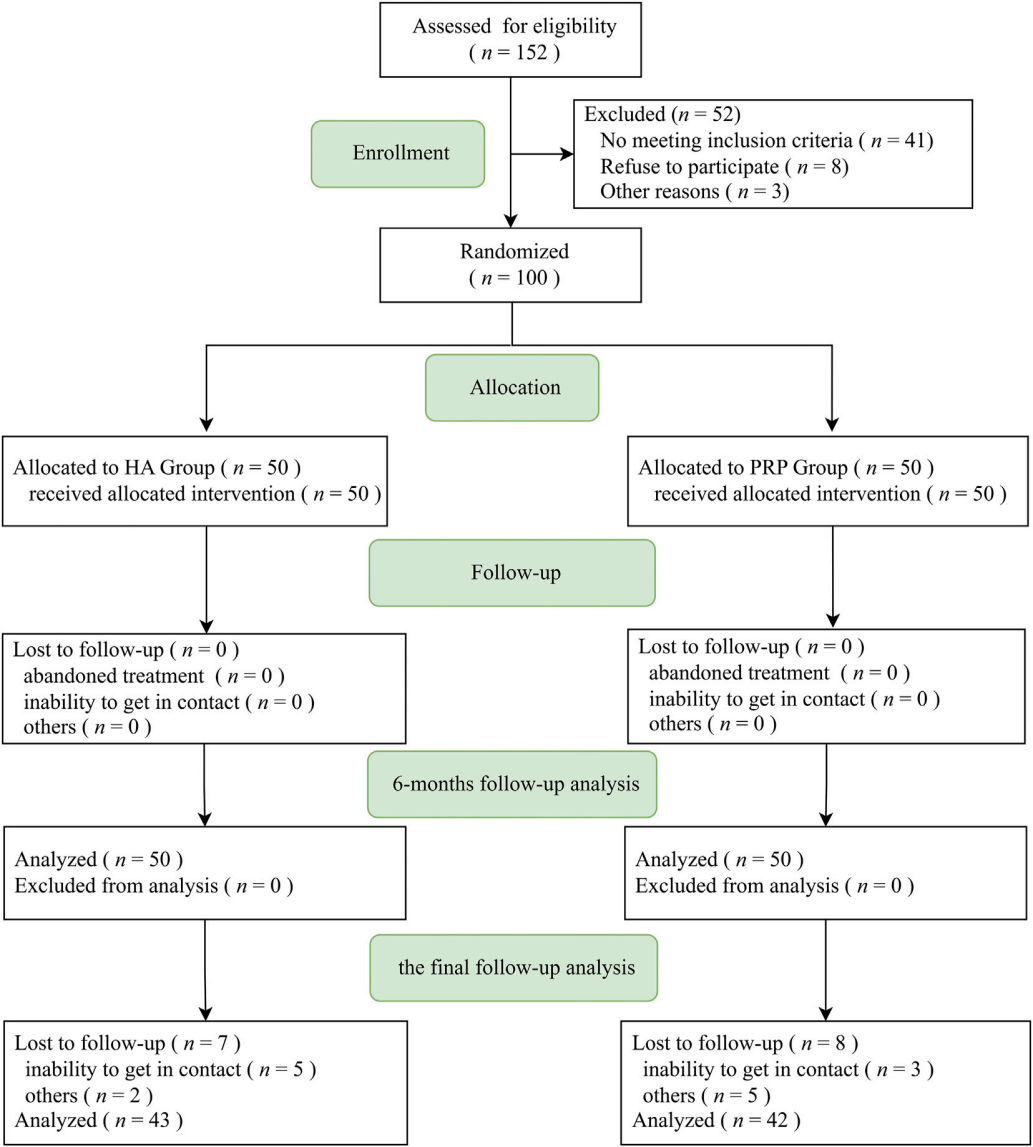
Consort flow chart. HA, hyaluronic acid; PRP, platelet-rich plasma.

152 patients were assessed for eligibility. 41 of them did not meet the inclusion criteria, eight of them declined to participate, and three of them were excluded because of other reasons (Figure 1). 100 patients were enrolled in the study and randomized into the two treatment groups in a random number table way: weekly intra-articular injections of leukocyte-rich PRP (LR-PRP) for 3 weeks or weekly administrations of high-molecular-weight HA (Sodium Hyaluronate injection; 25 mg/2.5 ml; ARTZ, Seikagaku Corporation).

### PRP preparation method

For each injection, a 40 ml blood sample was gathered from the median elbow vein and added with an anticoagulant. Two centrifugations were performed to obtain PRP: the first at 1,450 rpm to separate erythrocytes and then the second at 3,370 rpm to concentrate platelets for 10 min, respectively, which provided 5 ml of PRP divided into 1 ml and 4 ml. The former was sent to the laboratory for quality tests. Whereas the latter was transported to the injection room for treatment within 2 hours. Before the injection, PRP was activated by adding 10% calcium chloride. The platelet count was found to be 857.4 ± 151.2*10^9^/L, which was 6.1 times that in the preoperative peripheral blood. It should be mentioned that the preparation of the PRP was performed by a qualified laboratory physician blinded to clinical data.

### Follow-up Outcomes

**FIGURE 2:**
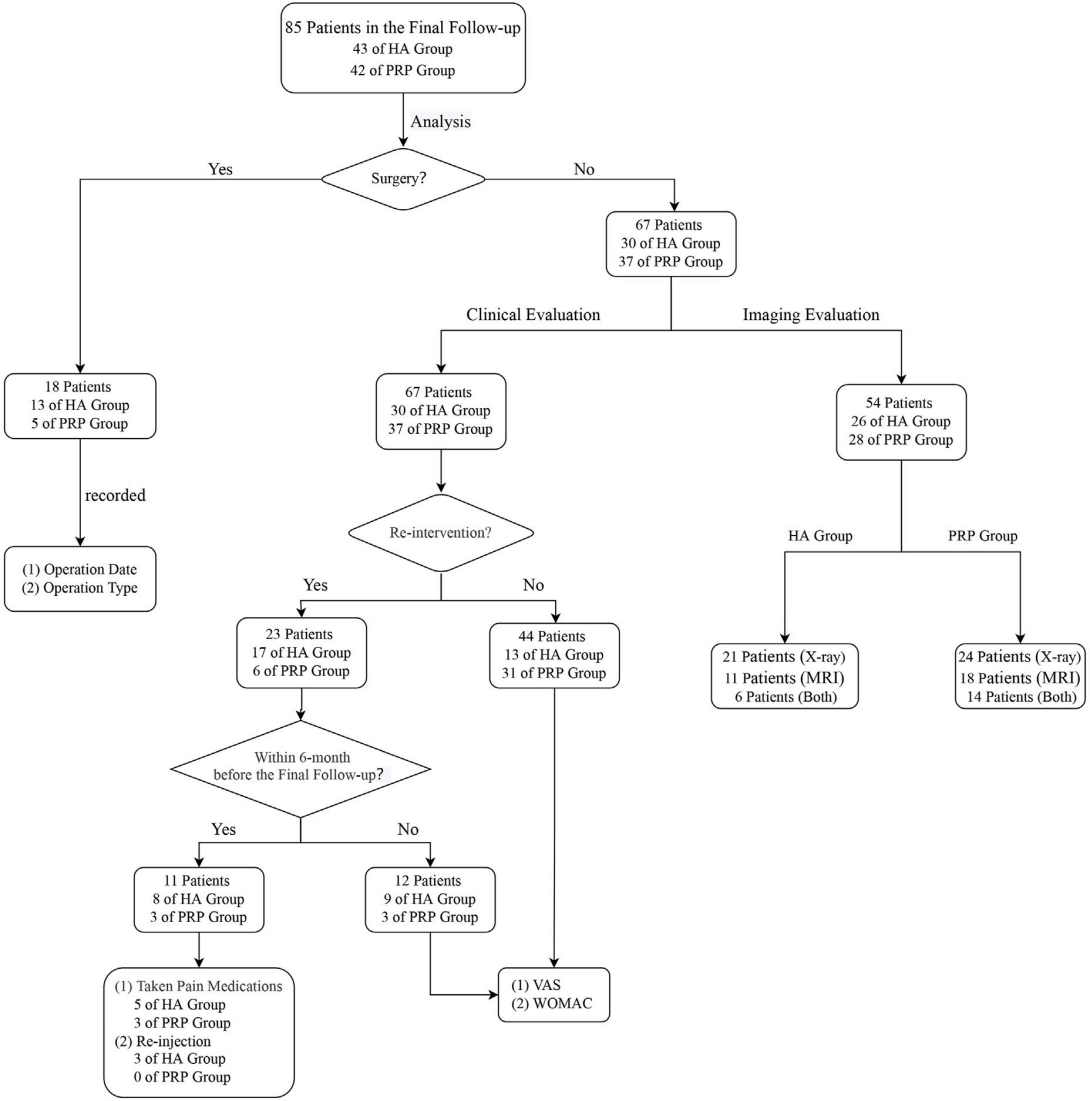
Analysis flowchart. HA, hyaluronic acid; PRP, platelet-rich plasma; MRI: Magnetic Resonance Imaging; VAS, visual analogue scale; WOMAC, Western Ontario and McMaster Universities Arthritis Index.

Outcomes were evaluated by an independent physician not involved injection procedure for pre-injection, post-3, 6, and a mean follow-up of 78.9-month (SD, 2.9 months) after the last injection. The final follow-up assessment procedures were carried out according to the following process (Figure 2).

Firstly, patients were classified as survival or failures based on their responses to the follow-up survey. Failure was defined as either needs arthroscopic knee surgery (AKS), unicompartmental knee arthroplasty (UKA), or total knee arthroplasty (TKA). For these patients, the date and type of surgical intervention were recorded. Others were interpreted as survival patients.

Secondly, for survival patients, the clinical and radiological outcomes were evaluated severally. Clinical assessments mainly included three aspects: re-intervention (pain medication usage or additional injections), the visual analog scale (VAS), and the Western Ontario and McMaster Universities Arthritis Index (WOMAC) (Bellamy, 2002). It should be pointed out that the VAS and WOMAC will not be evaluated when NSAIDs were employed in recent 6 months or injection therapies were given once. And the decision of performing surgery or re-intervention for further treatment was made by a blinded independent clinician.

Afterward, based on the patients’ compliance, either X-ray or 3.0T Magnetic Resonance Imaging (MRI) or both or neither, the radiological assessment was performed. Radiological assessment outcomes were generally expressed by K-L grade (Hochberg et al., 1995) or Cartilage Lesion Score (CaLS) (Alizai et al., 2014). K-L grade, a classic method to evaluate the severity of KOA, was estimated by X-ray. CaLS, a reproducible scoring system for cartilage lesions, was evaluated by MRI. It should be clarified that all the evaluation processes were assessed by two persons who were blinded to the intervention type.

In the end, satisfaction was assessed at five levels: disappointed, dissatisfied, neutral, satisfied, and very satisfied. Their answer was scored, respectively, one to five on a Likert scale (Xue et al., 2021). The number of complications and adverse events was also assessed.

### Statistical analysis

All analyses were performed with SPSS, version 25.0 (IBM Corp., NY, United States ). For continuous and discrete variables, they were reported as mean ± standard deviation (SD) or frequencies and percentages, respectively. Paired Student’s t-tests and independent sample tests were used to compare the intragroup and intergroup differences for normally distributed variables. The categorical data were analyzed by the chi-square or Fisher exact test. The knee survival was estimated following Kaplan-Meier analysis, with knee surgery for any cause as an event. The combined effect of the clinical and demographic characteristics on VAS and WOMAC was assessed by multiple regression analysis. A *p* value < 0.05 was deemed statistically significant.

## Results

### Baseline data

Of the 100 patients evaluated in the previous 6-month follow-up study, 85 were available for the mean 78.9-month follow-up, whereas 15 patients, for inability to get in contact and reluctance to participate, were lost at the time of the follow-up study. At midterm follow-up, there were seven patients in the HA group and eight in the PRP group lost to follow-up, respectively. There were 43 and 42 patients in the two groups. The baseline characteristics of the participants were homogeneous and comparable for all the parameters, which was detailed in Table 1.

**TABLE 1:** Characteristics of patients included in the two treatment groups.

	HA Group (*n* = 43)	PRP Group (*n* = 42)	*P*
Male (*n*/%)	9/20.9	11/26.2	NS
Age (years)	62.3 ± 8.9	64.9 ± 11.8	NS
BMI (kg/m^2^)	22.1 ± 3.6	23.4 ± 4.1	NS
Degenerative arthritis (*n*/%)	27/62.8	28/66.7	NS
Injection of left side (*n*/%)	25/58.1	23/54.8	NS
Follow-up (months)	79.2 ± 2.8	78.6 ± 2.9	NS

HA, hyaluronic acid; PRP, platelet-rich plasma; BMI, body mass index; NS, not significant.

#### Survival rate

**FIGURE 3:**
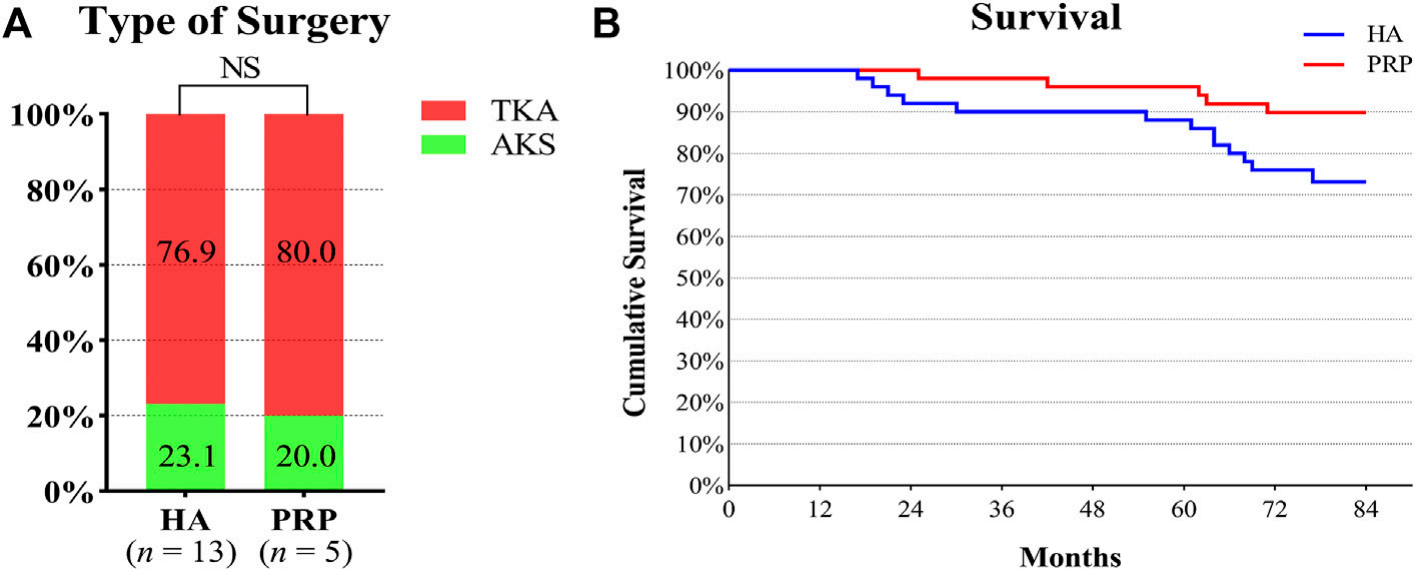
Type of surgery and survival rate in both treatment groups. **(A)** It showed the operation types of the two groups. The number in the columns indicated the proportion of operation types in each group. **(B)** Survival curve of the duration of the beneficial effect provided by the injective treatments. HA, hyaluronic acid; PRP, platelet-rich plasma; AKS, arthroscopic knee surgery; TKA, total knee arthroplasty; NS, not significant.

Before the last follow-up, 13 and five patients in the two groups underwent surgical treatment respectively. Three patients underwent AKS and 10 patients who underwent TKA in the HA group. One patient and four patients underwent AKS and TKA in the PRP group, respectively. There was no significant difference in surgery types (*p >* 0.05). With surgery for any reason as the endpoint, the cumulative survival rate of the PRP group was 90%, while that of the HA group was 74%, which was shown in Figures 3A, B.

### Clinical evaluation

The total re-intervention rate, re-intervention rate beyond the final follow-up 6 months, and within the final follow-up 6 months showed a significant increase in the HA group compared with the PRP group (*p* < 0.05, respectively). The comparative analysis showed no significant intergroup difference in the type of re-intervention within the final follow-up 6-month (*p >* 0.05, Table 2).

**TABLE 2:** Reintervention in the two treatment groups at the final follow-up.

	HA Group (*n* = 30)	PRP Group (*n* = 37)	*P*
Total Reintervention	17/56.7	6/16.2	**<0.01**
Reintervention beyond 6-month	9/30.0	3/8.1	**0.02**
Reintervention within 6-month	8/26.7	3/8.1	**0.04**
Taken Pain Medications	5/62.5	3/100.0	NS
Reinjection	3/37.5	0	-

HA, hyaluronic acid; PRP, platelet-rich plasma; BMI, body mass index; Boldface indicates *p* value < 0.05; NS, not significant.

The bold values indicate a significant difference between the two groups at the final follow-up.

**FIGURE 4:**
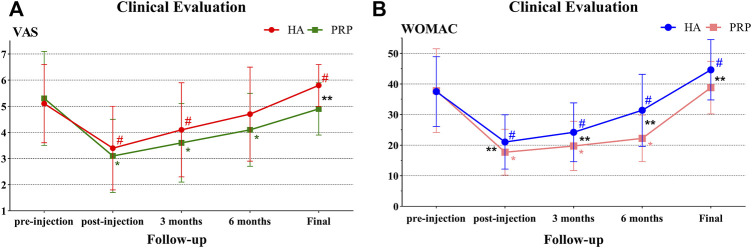
Clinical evaluation in both treatment groups. **(A)** VAS trend in both treatment groups at baseline, post-injection, 3 months, 6 months, and mean 78.9 months of follow-up. **(B)** WOMAC trend in both treatment groups at baseline, post-injection, 3 months, 6 months, and mean 78.9 months of follow-up. HA, hyaluronic acid; PRP, platelet-rich plasma; VAS, visual analogue scale; WOMAC, Western Ontario and McMaster Universities Arthritis Index; # indicates *p* value < 0.05 between the pre-injection with the follow-up in the HA group; * indicates *p* value < 0.05 between the pre-injection and the follow-up in the HA group; ** indicates *p* value < 0.05 between the two groups.

Unexpectedly, the VAS and WOMAC were higher than the baseline value at the final evaluation in the HA group and both significant differences were observed (*p* < 0.05). However, in the PRP group, the VAS decreased from 5.3 ± 1.8 at basal evaluation to 4.9 ± 1.0 (*p* > 0.05) at the final follow-up and the WOMAC increased from 37.8 ± 13.7 at basal evaluation to 38.8 ± 8.6 (*p* > 0.05) at the final follow-up. In particular, significant intergroup differences were reported in the VAS and WOMAC (5.8 ± 0.8 vs. 4.9 ± 1.0, *p* < 0.01; 44.6 ± 9.9 vs. 38.8 ± 8.6, *p* < 0.05; Table 3; Figure 4A, B).

**TABLE 3:** Comparison of VAS and WOMAC in the two treatment groups before and the final follow-up after the treatment.

	VAS		WOMAC	
	Pre-injection (*n* = 50)	Final follow-up	Pre-injection (*n* = 50)	Final follow-up
HA Group (*n* = 22)	5.1 ± 1.5	5.8 ± 0.8^#^	37.5 ± 11.4	44.6 ± 9.9^*^
PRP Group (*n* = 34)	5.3 ± 1.8	**4.9 ± 1.0**	37.8 ± 13.7	**38.8 ± 8.6**

HA, hyaluronic acid; PRP, platelet-rich plasma; VAS, visual analogue scale; WOMAC, Western Ontario and McMaster Universities Arthritis Index; # indicates *p* value < 0.01 between the pre-injection and the final follow-up in the HA, group; * indicates *p* value < 0.05 between the pre-injection and the final follow-up in the HA, group; Boldface indicates *p* value < 0.05 between the two groups at the final follow-up.

The bold values indicate a significant difference between the two groups at the final follow-up.

There were a number of factors that can impact VAS and WOMAC scores in our patients. Moreover, all these factors may also present associations between them. In order to identify which factors were statistically independent determinants, a multiple regression analysis was performed (Tables 4, 5). Type of treatment, sex, age, BMI, and K-L grade were taken into consideration to generate a multivariate linear regression equation. The results of the analysis showed that the type of treatment, age, and K-L grade served as independent determinants of VAS. Similarly, those variables independently determined WOMAC in KOA patients. Specifically, an inverse direct correlation of VAS and WOMAC was observed with the type of treatment. Patients treated with PRP had lower VAS and WOMAC compared with HA (B = -0.90, *t* = -4.96, *p* < 0.001; B = -6.47, *t* = -3.12, *p* = 0.003). In contrast, a significant direct correlation was observed with age and K-L grade. Elderly patients had higher VAS and WOMAC (B = 0.02, *t* = 2.18, *p* = 0.034; B = 0.31, *t* = 2.61, *p* = 0.012). Likewise, increased VAS and WOMAC were presented in patients with higher K-L grade (B = 0.68, *t* = 0.15, *p* < 0.001; B = 3.62, *t* = 2.22, *p* = 0.031). While there were no statistical differences in the effect of a different gender or BMI on VAS and WOMAC (*p* > 0.05, respectively).

**TABLE 4:** Multiple regression analysis of the combined influence of clinical characteristics and treatment of type variables on VAS in patients with KOA.

Variables	B	SE	β	*t*	*P*
Constant	4.18	1.08	-	3.87	**<0.001**
Type of treatment*	−0.90	0.19	−0.45	−4.96	**<0.001**
Sex*	−0.22	0.26	-0.09	-0.83	0.413
Age	0.02	0.01	0.23	2.18	**0.034**
BMI	0.01	0.03	0.05	0.45	0.654
K-L Grade*	0.68	0.15	0.48	4.64	**<0.001**

Model summary: r = 0.575; *p* < 0.001. Coefficient B indicates the number of units that the dependent variable increases by for each increase in the unit of the independent variable. SE, is the standard error. The coefficient *β* is the standardized coefficient B. VAS, visual analogue scale; KOA, knee osteoarthritis; BMI, body mass index; K-L; Kellgren-Lawrence; *The hyaluronic acid group, the male, and Kellgren-Lawrence grade I served as the control group, respectively. Boldface indicates the significant differences in *p* value.

The bold values indicate a significant difference in the dependent variable caused by the change in the independent variable.

**TABLE 5:** Multiple regression analysis of the combined influence of clinical characteristics and treatment of type variables on WOMAC in patients with KOA.

Variables	B	SE	β	*t*	*P*
Constant	29.67	12.10	−	2.45	**0.018**
Type of treatment*	−6.47	2.07	−0.349	−3.12	**0.003**
Sex*	−5.52	2.96	-0.23	−1.87	0.068
Age	0.31	0.12	0.33	2.61	**0.012**
BMI	0.26	0.29	0.11	0.87	0.389
K-L Grade*	3.62	1.64	0.27	2.22	**0.031**

Model summary: r = 0.328; *p* < 0.001.

Coefficient B indicates the number of units that the dependent variable increases by for each increase in the unit of the independent variable. SE, is the standard error. The coefficient *β* is the standardized coefficient B. WOMAC, Western Ontario and McMaster Universities Arthritis Index; KOA, knee osteoarthritis; BMI, body mass index; K-L; Kellgren-Lawrence; *The hyaluronic acid group, the male, and Kellgren-Lawrence grade I served as the control group, respectively. Boldface indicates the significant differences in *p* value.

The bold values indicate a significant difference in the dependent variable caused by the change in the independent variable.

### Imaging evaluation

**FIGURE 5:**
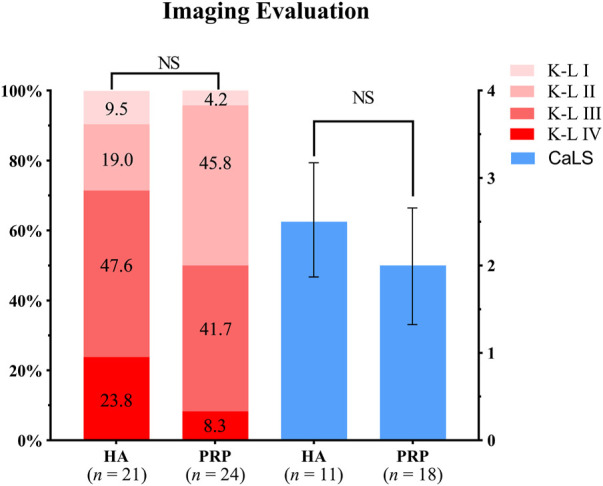
Imaging evaluation in both treatment groups. The left two columns showed the Kellgren-Lawrence grades of the two groups, of which the proportions were indicated by the number in the columns. The right showed the Cartilage Lesion Score of the two groups. HA, hyaluronic acid; PRP, platelet-rich plasma; K-L, Kellgren-Lawrence; CaLS, Cartilage Lesion Score; NS, not significant.

**FIGURE 6:**
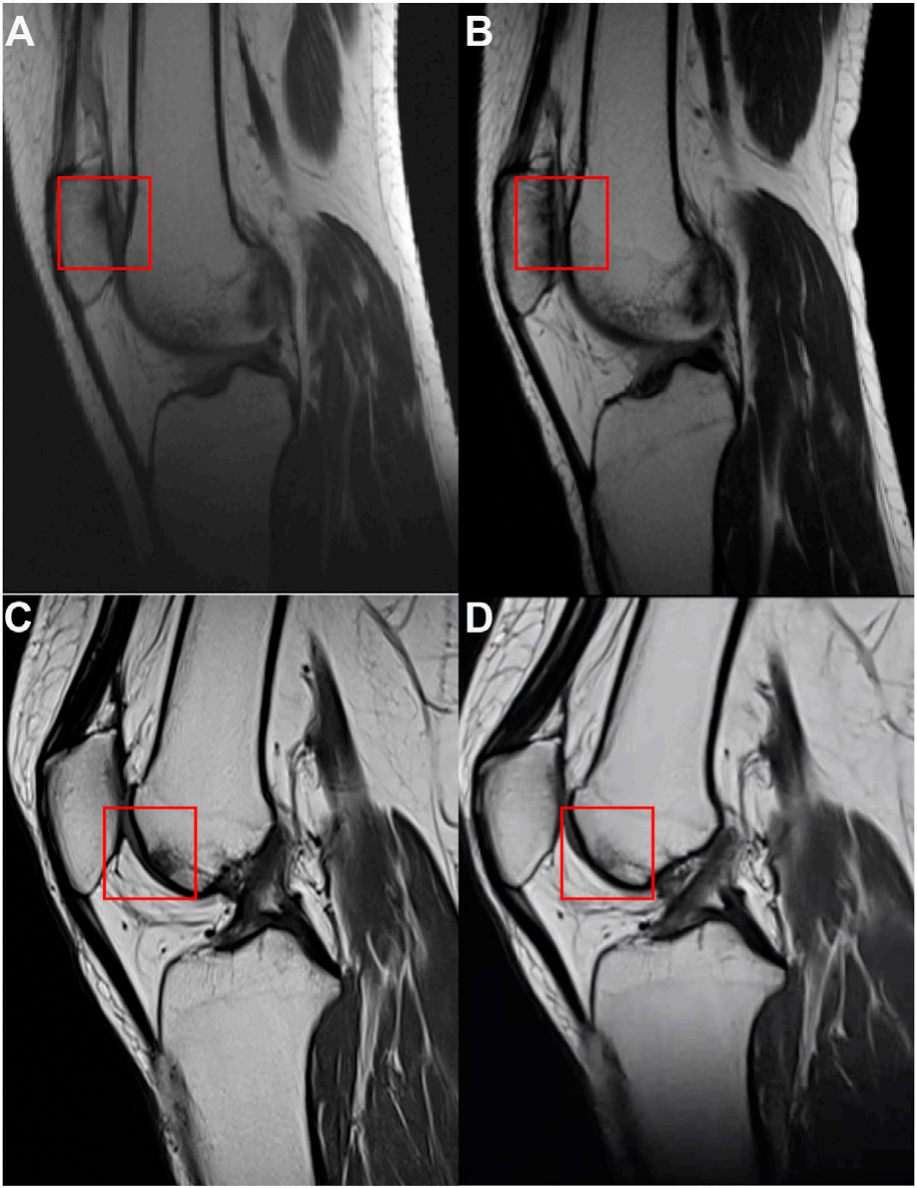
The MRI of the case in two groups. **(A)** MRI of a case before injection in the hyaluronic acid group. **(B)** MRI of the case at the final follow-up in the hyaluronic acid group. **(C)** MRI of a case before injection in the platelet-rich plasma group. **(D)** MRI of the case at the final follow-up in the platelet-rich plasma group. The comparisons of bone marrow lesion were shown by the red square.

In the PRP group, 26 patients participated in the imaging evaluation, of which 21 patients took X-rays, 11 patients underwent MRI examination, and six patients underwent both. Whereas in the HA group, 28 patients participated in the imaging evaluation, of which 24 patients took X-rays, 18 patients underwent MRI, and 14 patients underwent both. No difference was found for the K-L grade between the two groups (*p* > 0.05, Figure 5), while the rate of K-L III or IV grade in the HA group was higher than that of the PRP group. The mean CaLS in the HA and the PRP groups were 2.5 (SD 0.65) and 2.0 (SD 0.67), respectively, which was not significantly different (*p* > 0.05, Figure 5). However, a couple of promising phenomena have been discovered in the MRI. The comparison of bone marrow lesion area was shown in Figure 6 for three representative cases before injection and at the final follow-up in two groups.

#### Satisfaction and complication

There were 24 (55.8%) and 33 (78.6%) patients in the HA and the PRP group respectively, which accorded with the definition of having a satisfactory outcome. It exhibited a statistically significant difference between groups (*p* < 0.05). We did not have any case of infection, poor healing, or neurological lesion in the final follow-up.

## Discussion

Osteoarthritis is a chronic degenerative disease, resulting in the progressive loss of articular cartilage, with complicated pathophysiology (Shahid and Kundra, 2017; Barnett, 2018; Hunter and Bierma-Zeinstra, 2019). In General, cartilage is avascular which gives rise to its lack of inherent healing potential. Without intervention, the progression of KOA from early to late stage is rapid, for which the only treatment is surgery, and the symptoms can extremely impair people’s quality of life. It is essential to put off the appearance of the operation, which is attributable to the irreversibility of the procedure, the limited life of the prosthesis, and the frequency of postoperative pain. Fortunately, various measures have been developed and worked (Barnett, 2018; Hunter and Bierma-Zeinstra, 2019; Deyle et al., 2020; Sharma, 2021). However, there has become a shift from primarily pharmacologic therapy to regenerative cellular therapy, owing to the unsatisfactory effect, limited benefits, and the risk of adverse events of the former in recent years (Lawrence et al., 2008; Bennell et al., 2017). Surprisingly, the application of PRP has been demonstrated and proven to be beneficial for repairing cartilage lesions or osteochondral defects *in vitro* (Sun et al., 2010). And the subsequent clinical benefits corroborated the effectiveness of PRP therapies, with encouraging patient outcomes reported (Dai et al., 2017; Simental-Mendía et al., 2019; Li et al., 2021; Sánchez et al., 2021).

Currently, the therapeutic effect of PRP is related to various factors, such as preparation, formulation, and frequency of injection (Simental-Mendía et al., 2019; El-Husseiny et al., 2021; Hong et al., 2021). The optimal PRP application proposal is controversial as a result of the various results in studies. There was no guiding standardization for PRP preparation in the literature when the trial was developed. Therefore, our study applied the mainstream PRP treatment proposal, double-spin preparation, leukocyte-rich formulation, and session once a week for 3 weeks, to achieve better effects. At a minimum 7-year follow-up, there were no significant differences in the imaging evaluation between the two groups (*p >* 0.05). However, on the one hand, there were lower re-intervention rates, VAS, and WOMAC (*p* < 0.05, respectively) in the PRP group. On the other hand, patients in the PRP group had a higher survival rate and satisfaction than those in the HA group (78.6% vs 55.8%, *p* < 0.05). In addition, based on the regression analysis, we concluded that the influence of treatment, age, and K-L grade on VAS and WOMAC were statistically independent determinants (*p* < 0.05, respectively).

In light of our outcomes, there were the following points worthy of attention. To begin with, the result of higher survival in the group of PRP was consistent with the previous study (Sánchez et al., 2021), which indicated that the application of PRP in KOA patients was a treatment that could delay knee operation. It is generally believed that TKA and AKS are closely related to pain and dysfunction of the knee. Similarly, in one of our results, VAS and WOMAC were smaller in the PRP group, which manifested that PRP had more superiorities than HA in alleviating pain and ameliorating joint function. HA, a viscosupplementation of non-sulfated glycosaminoglycan, whose natural form is present in healthy joint fluid, has been extensively used as an adjunct in cartilage repair (Russo et al., 2016). It can modulate inflammation inhibiting matrix metalloproteinases (MMPs) *in vitro* study (Prasadam et al., 2013). Comparatively, numerous studies (Sampson et al., 2010; Wu et al., 2018; Qi et al., 2021; Yang et al., 2021; Zhao et al., 2021) have shown the effectiveness of PRP in the treatment of KOA through various signaling transduction pathways. The PRP can suppress levels of inflammatory factors tumor necrosis factor-α, interleukin-1β, and interleukin-6, and protect chondrocytes from IL-1β-induced chondrocyte apoptosis and extracellular matrix degradation by inhibiting hypoxia-inducible factor 2*α* (Yang et al., 2021; Zhao et al., 2021). Briefly, the PRP itself releases the “cytokine cocktail” of the healing cascade, containing a plurality of growth factors, which can initiate the chemotaxis of immunocompetent cells, inflammation, angiogenesis, and as a consequence the process of synthesis of the extracellular matrix and tissue remodeling (Andia and Maffulli, 2018; Kaminski et al., 2019). The PRP acts at various levels for joint homeostasis. Paradoxically, compared with the HA group, there was no advantage in the imaging evaluation in the PRP group, including K-L grade and CaLS, which hinted that the cartilage repair effect of PRP in mid-term clinical outcomes required to be further determined and findings from *in vivo* and *in vitro* studies could not be directly translated to clinical practice.

Furthermore, age is one of the most evident risk factors for OA (Hunter and Bierma-Zeinstra, 2019), of which severity is usually ranked by K-L grade with X-ray. Undoubtedly, there were significant correlations between the above two points with VAS or WOMAC. On the basis of that, it is crucial to pay attention to the necessity of treating KOA as soon as possible. This was in line with results from Saraf et al. (Saraf et al., 2022). Unpredictably, the change in BMI or gender did not cause statistical differences in our results, while it routinely was assigned as a moderate to strong risk factor with strong pieces of evidence (Silverwood et al., 2015). From our perspective, it resulted from the imbalance of sex ratio (10 vs 46) when the pain indicators were scored. With respect to BMI, although its change did not contribute to the statistical difference of knee pain and functional scores, of which the tendency was in compliance with the evidence. Namely, as BMI increased, so did VAS and WOMAC. It was our view that this was caused by the coincidence of many variables. Nevertheless, it should be pointed out that several studies (Akhlaque et al., 2020; Saraf et al., 2022) have also put forward the same results, hence the relevance of BMI with outcomes warrant further consideration.

The findings of this study have to be seen in the light of some limitations. Firstly, many patients were lost to follow-up because of the long follow-up interval. Secondly, due to the discrepancy in the cost of treatment, the study was single-blinded. Thirdly, this study’s sample sizes were small, further limiting the reliability of the results. Then, considering the long follow-up interval, the patients may have also undergone some physical therapies or other treatments, which have been forgotten. We can not conclude that the PRP injection was the sole contributor to patient-reported improvements in pain and function. Eventually, due to the lack of PRP guidelines during the study period, the cellular analysis and growth factors determination were not performed, which should be regarded as another limit of validity and reproducibility of the data in the present study. Numerous high-quality, large-sample, and long-term studies are required to verify the effects of PRP injection in KOA.

## Conclusion

These mid-term results indicated that PRP was more effective than HA in intervention rates, VAS, and WOMAC, although there were no significant differences in the survival rate and imaging evaluation between the two groups. Furthermore, patients treated with PRP were associated with higher satisfaction compared with HA. Therefore, additional clinical studies are required before PRP injections can be used more extensively.

## Data Availability

The raw data supporting the conclusion of this article will be made available by the authors, without undue reservation.
